# Identification and validation of a novel ferroptosis-related gene signature for prognosis and potential therapeutic target prediction in cholangiocarcinoma

**DOI:** 10.3389/fimmu.2022.1051273

**Published:** 2023-01-17

**Authors:** Apiwit Sae-fung, Apiwat Mutirangura, Siriporn Jitkaew

**Affiliations:** ^1^ Graduate Program in Clinical Biochemistry and Molecular Medicine, Department of Clinical Chemistry, Faculty of Allied Health Sciences, Chulalongkorn University, Bangkok, Thailand; ^2^ Department of Anatomy, Faculty of Medicine, Center of Excellence in Molecular Genetics of Cancer and Human Diseases, Chulalongkorn University, Bangkok, Thailand; ^3^ Department of Clinical Chemistry, Faculty of Allied Health Sciences, Chulalongkorn University, Bangkok, Thailand; ^4^ Age-Related Inflammation and Degeneration Research Unit, Department of Clinical Chemistry, Faculty of Allied Health Sciences, Chulalongkorn University, Bangkok, Thailand

**Keywords:** ferroptosis, cholangiocarcinoma, gene signature, prognosis, risk score

## Abstract

Cholangiocarcinoma (CCA) is a highly heterogeneous and aggressive malignancy of the bile ducts with a poor prognosis and high mortality rate. Effective targeted therapy and accurate prognostic biomarkers are still lacking. Ferroptosis is a form of regulated cell death implicated in cancer progression and has emerged as a potential therapeutic target in various cancers. However, a comprehensive analysis of ferroptosis-related genes (FRGs) for predicting CCA prognosis and therapeutic targets and determining the role of ferroptosis in CCA remain to be performed. Here, we developed a prognostic FRG signature using a least absolute shrinkage and selection operator Cox regression analysis in a training cohort. We then validated it using four independent public datasets. The six-FRG signature was developed to predict CCA patient survival, stratifying them into low-risk and high-risk groups based on survival time. Significantly, the high-risk CCA patients had shorter overall survival. A receiver operating characteristic curve analysis further confirmed the prognostic FRG signature’s strong predictive ability, indicating that it was an independent prognostic indicator for CCA patients. Furthermore, the high-risk group was associated with fluke infection and high clinical stages. Cancer-associated fibroblast (CAF) score and CAF markers were significantly higher in the high-risk group than the low-risk group. Moreover, our FRG signature could predict immune checkpoint markers for immunotherapy and drug sensitivity. The mRNA expression levels of the six-FRG signature was validated in 10 CCA cell lines and dividing them into low-risk and high-risk groups using the FRG signature. We further showed that high-risk CCA cell lines were more resistant to ferroptosis inducers, including erastin and RSL3, than the low-risk CCA cell lines. Our study constructed a novel FRG signature model to predict CCA prognoses which might provide prognostic biomarkers and potential therapeutic targets for CCA patients. Ferroptosis sensitivity in high-risk and low-risk CCA cell lines suggests that ferroptosis resistance is associated with high-risk group CCA. Therefore, ferroptosis could be a promising therapeutic target for precision therapy in CCA patients.

## 1 Introduction

Cholangiocarcinoma (CCA/CHOL) is a highly heterogeneous malignancy originating from epithelial bile ducts at any level of the bile duct tree. Its incidence rate has significantly increased worldwide, with higher prevalence in Asian countries over the past few years (1). Due to a lack of effective early diagnosis, most CCA patients are usually diagnosed at advanced stages where surgical resection cannot be performed (2, 3). While, therapeutic options for CCA patients are increasing, such as chemotherapy, targeted therapy, and immunotherapy, their overall prognosis remains unsatisfactory (4–6). Therefore, identifying novel predictive models, accurate prognostic biomarkers, and novel therapeutic targets are urgently required to improve overall survival of CCA patients.

Ferroptosis is a novel regulated form of cell death that relies on iron overload, accumulation of reactive oxygen species (ROS) and polyunsaturated fatty acids (PUFA), and phospholipid peroxidation (7–9). System ${\text{X}}_{\text{c}}^{-}$ and glutathione peroxidase 4 (GPX4) are key regulators in the glutathione pathway that control the ferroptosis mechanism. Erastin and RAS-selective lethal (RSL3) can induce ferroptosis by inhibiting system ${\text{X}}_{\text{c}}^{-}$ and GPX4 activity, respectively (9–11). Recent studies have shown that ferroptosis represents a novel target for efficient therapeutic strategies to overcome treatment resistance in several cancers (12). In addition, ferroptosis-related genes (FRGs) and ferroptosis signaling dysregulation might be associated with cancer patient prognosis. However, the role of ferroptosis in CCA progression and prognosis remain unknown. In addition, very few studies have explored the therapeutic applications of ferroptosis for CCA patients. Therefore, discovering key prognostic FRGs predicting CCA prognosis and clinical outcomes is urgently needed since they might act as novel biomarkers and therapeutic targets for CCA patients.

This study obtained RNA expression profiles and clinical data from two cohorts in the Gene Expression Omnibus (GEO) database and developed a six-FRG signature. We validated our six-FRG signature for CCA prognosis prediction in four independent cohorts in GEO, The Cancer Genome Atlas (TCGA), the European Bioinformatics Institute (EMBL-EBI) database, and the National Omics Data Encyclopedia (NODE) database. Our six-FRG signature was used to stratify CCA patients into two groups, confirming their prognosis prediction. In addition, these two patient groups differed in their functional and biological processes, immune cell infiltration, cancer-associated fibroblast (CAF) abundance, and drug sensitivity. Moreover, we confirmed the mRNA expression levels of these six-FRG signature in a panel of CCA cell lines, dividing them into two groups based on their mRNA expression levels for these six-FRG signature. We further investigated the sensitivity of these CCA cell lines to ferroptosis inducers, including erastin and RSL3.

## 2 Materials and methods

### 2.1 Data collection and processing

**Figure 1:**
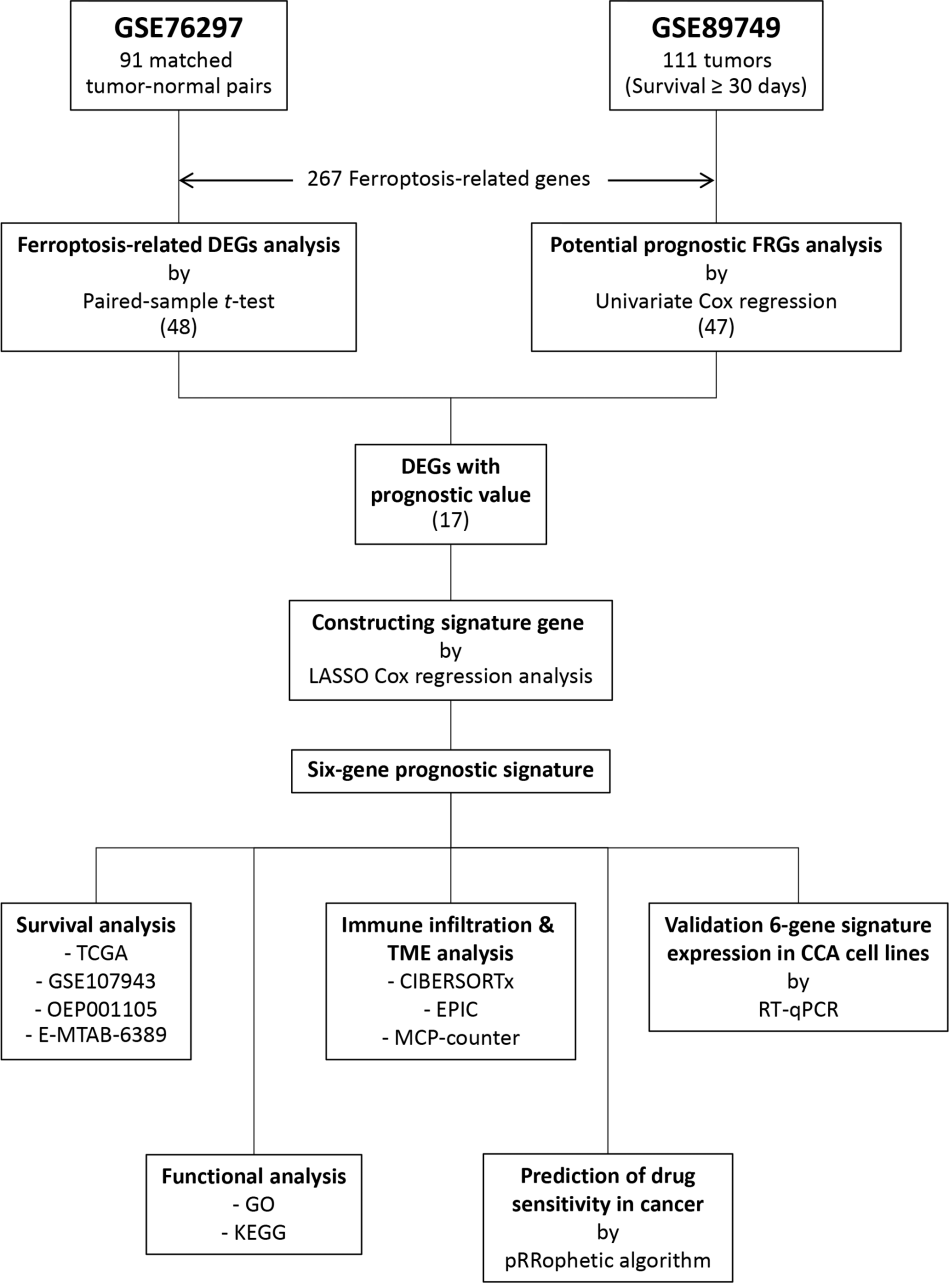
Flow chart of the study.

A flow chart describing the data collection and analysis process is provided in **Figure 1**. Five public RNA expression and clinical information were downloaded from four platforms. The TCGA-CHOL dataset was downloaded from the University of California at Santa Cruz (UCSC) Xena platform (https://xena.ucsc.edu/). The E-MTAB-6389 dataset was downloaded from the European Bioinformatics Institute (EMBL-EBI) database (https://www.ebi.ac.uk/). The OEP001105 dataset reported by a previous study (13) was downloaded from The National Omics Data Encyclopedia (NODE) database (https://www.biosino.org/node/). The GSE76297, GSE89749, and GSE107943 datasets were downloaded from the Gene Expression Omnibus (GEO) database (https://www.ncbi.nlm.nih.gov/geo/). The GSE89749 dataset’s clinical information was obtained from previous study (14). In total, 267 FRGs were identified from published studies (15, 16) and the FerrDb database (http://www.zhounan.org/ferrdb/) (17). CCA patient characteristics in all datasets are summarized in **Supplementary Table S1**.

### 2.2 Identification of ferroptosis-related differentially expressed and prognostic genes

Differentially expressed genes (DEGs) were identified in the GSE76297 cohort, which includes 91 pairs of tumor and non-tumor tissues using a paired-sample *t*-test with a |log_2_(fold change)| > 1 and *p*-value < 0.0001. A univariate Cox regression analysis was performed to screen out prognostic genes associated with overall patient survival in the GSE89749 cohort. This cohort includes 111 patients of which only those with an overall survival ≥ 30 days were included to ensure this study’s reliability. Genes with a *p*-value < 0.05 were considered prognostic genes. Finally, overlapping DEGs and prognostic genes were identified as candidate genes using a Venn diagram. A protein-protein interaction (PPI) network analysis was performed on candidate genes by the STRING database (https://www.string-db.org/) (18).

### 2.3 FRG signature construction

The GSE89749 cohort was used as a training cohort to construct an FRG signature. A least absolute shrinkage and selection operator (LASSO) Cox regression analysis was used to develop the FRG signature from candidate genes using the R statistical software’s “*glmnet*” package. Then, the following formula was used to calculate a ferroptosis-related risk score for each patient: risk score = (Exp.Gene_1_ × Coef.Gene_1_) + (Exp.Gene_2_ × Coef.Gene_2_) + … + (Exp.Gene_n_ × Coef.Gene_n_), where Exp.Gene is the expression of FRG signature genes and Coef.Gene is the regression coefficient obtained from the LASSO Cox regression analysis. The 111 patients in the GSE89749 cohort were stratified into low-risk and high-risk groups based on the median risk score. Kaplan-Meier and log-rank test were used to compare patient survival between risk groups using R’s “*survminer*” package. A time-dependent receiver operating characteristic (ROC) curve was used to predict the FRG signature’s specificity and sensitivity for predicting patient survival at 1, 3, and 5 years using R’s “*survivalROC*” package. The FRG signature’s prognostic potential was validated using the OEP001105, E-MTAB-6389, GSE107943, and TCGA-CHOL datasets.

### 2.4. Independent prognostic value analysis

A univariate Cox regression analysis was used to evaluate the prognostic value of the FRG signature and other clinical characteristics, including age, liver fluke infection, sex, and stage. A multivariate Cox regression analysis was performed to evaluate whether the FRG signature was an independent prognostic factor. Each variable’s hazard ratio (HR) and 95% confidence interval (CI) were calculated, with *p*-value < 0.05 considered statistically significant. Moreover, Fisher’s exact test was used to assess differences in clinical characteristics between low-risk and high-risk groups.

### 2.5 Functional gene set enrichment analysis

A GSEA was performed in the training cohort using GSEA software to identify functions and pathways enriched between low-risk and high-risk groups based on Gene Ontology (GO; c5.go.v7.5.1.symbols.gmt) and Kyoto Encyclopedia of Genes and Genomes (KEGG; c2.cp.kegg.v7.5.1.symbols.gmt). Gene sets with a | normalized enrichment score (NES)| > 1, *p*-value < 0.05, and false discovery rate (FDR) < 0.25 were considered statistically significant.

### 2.6 Immune cell infiltration and tumor microenvironment analysis

The CIBERSORTx algorithm (https://cibersortx.stanford.edu/) (19) was used to analyze the immune cell fractions of 22 immune cell types in the training cohort. Moreover, the MCP-counter (20) and EPIC (http://epic.gfellerlab.org/) (21) algorithms were used to estimate CAF abundance.

### 2.7 Drugs sensitivity and immunotherapy prediction

Differences in drugs sensitivity between the two patient groups were estimated by comparing half-maximal inhibitory concentration (IC_50_) using R’s “*pRRophetic*” package and the Genomics of Drug Sensitivity in Cancer (GDSC) database (https://www.cancerrxgene.org/) (22). Furthermore, the differential expression of common immune checkpoints in low-risk and high-risk groups was examined to predict potential immunotherapy targets.

### 2.8 Cell culture

The human immortalized non-tumor cholangiocyte cell line (MMNK-1) and CCA cell lines (CCLP-1, HuCCT-1, KKU-055, KKU-100, KKU-213, KKU-214, RBE, and TFK-1) were obtained from the Japanese Collection of Research Bioresources (JCRB) Cell Bank (Osaka, Japan). The HuCCA-1 and RMCCA-1 CCA cell lines were developed from Thai patients with CCA (23, 24). All cell lines were grown in Dulbecco’s modification of Eagle’s medium (DMEM; HyClone Laboratories, Logan, UT, USA) supplemented with 10% fetal bovine serum (Sigma-Aldrich, St Louis, MO, USA) and 1% Penicillin–Streptomycin (HyClone Laboratories) and were cultured in a humidified incubator at 37°C with 5% carbon dioxide. All cell lines were tested to be negative for mycoplasma contamination.

### 2.9 Reverse transcription-quantitative PCR

Total RNA was extracted from the cells using GENEzol Reagent (Geneaid Biotech, Taiwan). Then, 1 μg of RNA was reverse-transcribed using a Maxime RT PreMix Kit (iNtRON Biotechnology, Seongnam-si, Gyeonggi-do, Republic of Korea). RT-qPCR was performed using iTaq universal SYBR Green Supermix (Bio-Rad, Hercules, CA, USA) following the manufacturer’s instructions. All primers used in this study are listed in **Supplementary Table S2**. Relative expression in CCA cell lines was normalized to MMNK-1 cell line using the 2^-ΔΔCt^ method with β-actin (ACTB) as an internal control. Moreover, average 2^-ΔCt^ values were used as each gene’s values to classify CCA cell lines. Then, the expression values were transformed to *z*-score in each gene of all CCA cell lines. These *z*-score expression values were used to calculate risk scores in CCA cell lines which were then stratified into low-risk and high-risk FRG groups.

### 2.10 Treatment and cell viability assay

Erastin and RSL3 were obtained from ApexBio Technology (Boston, MA, USA). Two CCA cell lines groups based on the FRG risk groups, including CCLP-1, KKU-214, RBE, and RMCCA-1 were seeded in 96-well plates and incubated in a humidified incubator at 37°C with 5% carbon dioxide for 24 h. The cells were treated for 48 h using a two-fold serial dilution method with erastin concentrations of 0.3125, 0.625, 1.25, 2.5, 5, 10, 20, and 40 μM or RSL3 concentrations of 7.8125, 15.625, 31.25, 62.5, 125, 250, 500, and 1000 nM. Cell viability was determined using 3-(4,5-dimethylthiazol-2-yl)-2,5-diphenyl-2H-tetrazolium bromide (MTT) assay after 48 h of treatment. Briefly, 10 μl of MTT reagent was added to each well and incubated in a humidified incubator for 2 h. Next, the supernatant was removed, and 100 μl of dimethyl sulfoxide (DMSO) was added to each well. Then, cell viability was determined at 570 nm using a microplate reader and the percentage of viable cells was calculated and normalized to the DMSO vehicle control. The IC_50_ of erastin and RSL3 was calculated and compared between two CCA cell line groups. Three-independent experiments were performed with triplicate samples.

### 2.11 Statistical analysis

All data were analyzed using the R statistical software (version 4.1.0) or SPSS (version 22.0, IMM Corp; Armonk, NY, USA). Wilcoxon or Student’s *t*-tests were used to assess differences between groups. The log-rank test was used to assess differences in survival between low-risk and high-risk groups, and a Kaplan-Meier curve was used to visualize patient survival. Pearson’s correlation coefficient (*r*) was used in all correlation analyses. All results with *p*-value < 0.05 were considered statistically significant (*p < 0.05, **p< 0.01, ***p < 0.001, ****p < 0.0001).

## 3 Results

### 3.1 Candidate gene identification

**Figure 2:**
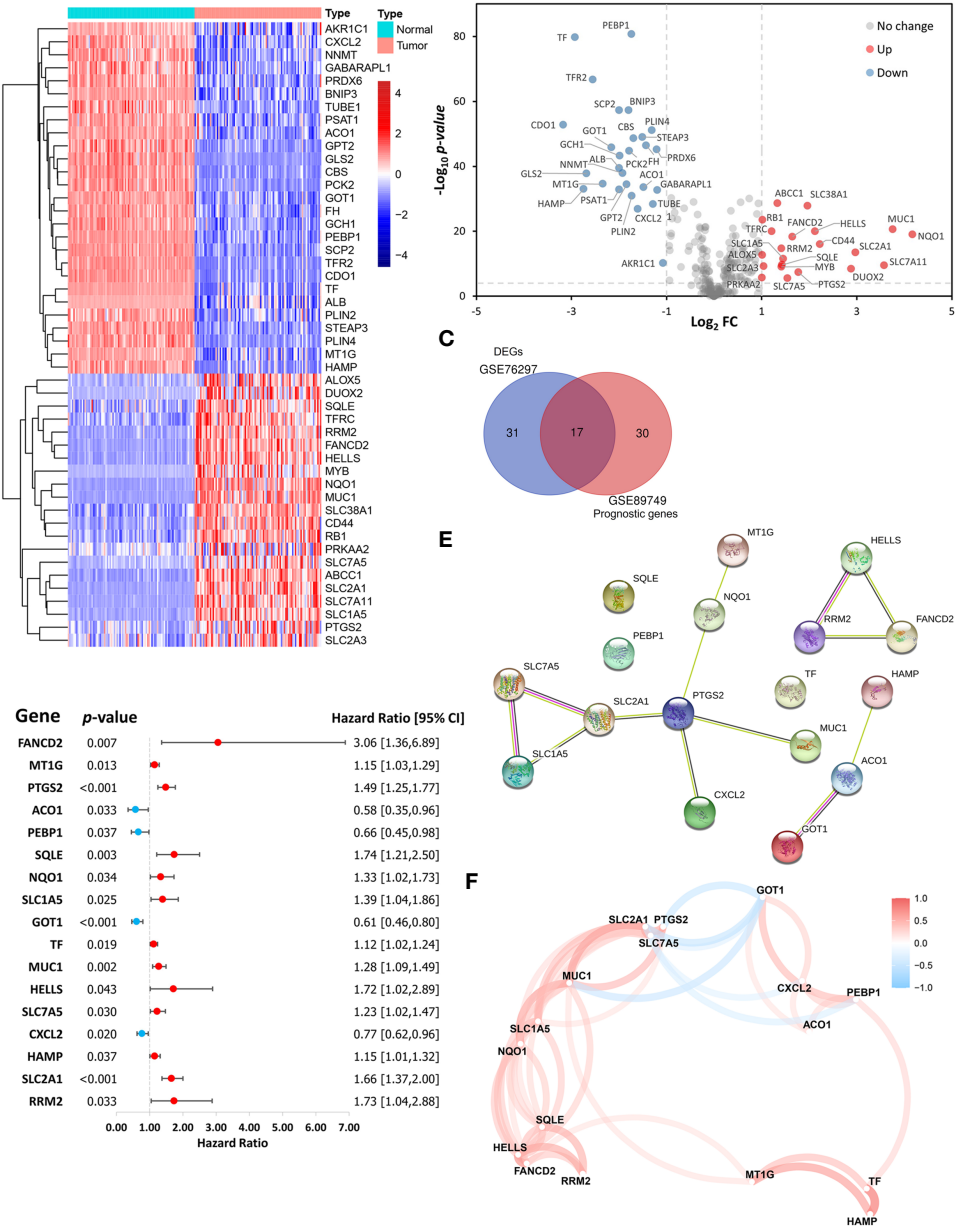
Identification of ferroptosis-related candidate genes. **(A)** Heatmap and **(B)** volcano plot showing the expression levels and distribution of differentially expressed genes (DEGs) in cholangiocarcinoma (CCA) tissues and normal tissues in the GSE76297 cohort. **(C)** A Venn diagram showing the intersection between DEGs and prognostic genes. **(D)** A forest plot showing the hazard ratios of candidate genes. **(E)** A protein-protein interaction (PPI) network of the candidate genes in the STRING database. **(F)** A correlation network of the candidate genes.

This study included 267 FRGs which are listed in **Supplementary Table S3**. Forty-eight ferroptosis-related DEGs were identified between paired tumor and non-tumor tissues in the GSE76297. Heatmap and volcano plots visualized their expression and distribution among samples, with 21 upregulated and 27 downregulated (**Figures 2A, B**). One hundred eleven patients with overall survival ≥ 30 days from the GSE89749 dataset were used a univariate Cox regression analysis to identify prognostic genes. The 47 prognostic genes associated with survival are listed in **Table 1**. A Venn diagram identified 17 intersecting genes (candidate genes) among the 48 ferroptosis-related DEGs and 47 prognostic genes (**Figure 2C**). The univariate Cox regression results for these 17 candidate genes were visualized in a forest plot (**Figure 2D**). Four were protective factors (*ACO1*, *PEBP1*, *GOT1*, and *CXCL12*), and 13 genes were risk factors (*FANCD2*, *MT1G*, *PTGS2*, *SQLE*, *NQO1*, *SLC1A5*, *TF*, *MUC1*, *HELLS*, *SLC7A5*, *HAMP*, *SLC2A1*, and *RRM2*) in CCA patients. The PPI network indicated associations between 17 candidate genes (**Figure 2E**). Correlations among these genes are shown by a correlation network (**Figure 2F**).

**Table 1:** Univariate Cox regression analysis of ferroptosis-related genes in GSE89749 cohort.

Gene	Coefficient	*p*-value
CS	-1.2200	0.0013
FANCD2	1.1184	0.0069
GSS	0.6494	0.0307
MT1G	0.1409	0.0127
PTGS2	0.3962	<0.0001
SAT1	0.3975	0.0300
ACO1	-0.5487	0.0334
ACACA	0.6612	0.0317
PEBP1	-0.4156	0.0374
SQLE	0.5543	0.0028
NQO1	0.2857	0.0336
SLC1A5	0.3307	0.0249
GOT1	-0.4960	0.0003
ACSF2	0.4344	0.0014
NOX4	0.3524	0.0198
FLT3	-1.2662	0.0445
HRAS	0.4983	0.0423
TF	0.1166	0.0193
BECN1	0.6708	0.0283
WIPI2	-1.2005	0.0192
MAPK3	0.5606	0.0076
YY1AP1	-1.2359	0.0225
HSF1	0.8390	0.0109
MUC1	0.2436	0.0023
HELLS	0.5399	0.0428
SCD	0.2713	0.0441
STAT3	0.8531	0.0045
MTOR	0.5988	0.0361
TP63	0.5130	0.0187
ISCU	-0.5828	0.0123
LAMP2	-0.4613	0.0052
UBC	0.9236	0.0245
OXSR1	0.7610	0.0016
DDIT4	0.3507	0.0059
DDIT3	-0.4767	0.0189
SLC7A5	0.2031	0.0301
TRIB3	-0.3108	0.0092
ZFP69B	-1.3924	0.0270
GDF15	-0.2765	0.0038
CXCL2	-0.2605	0.0203
HAMP	0.1417	0.0370
MAP3K5	0.5430	0.0013
SLC2A1	0.5045	<0.0001
RRM2	0.5508	0.0333
CAPG	0.6259	0.0011
AURKA	0.3492	0.0125
PRDX1	0.5882	0.0137

### 3.2 FRG signature construction

**Figure 3:**
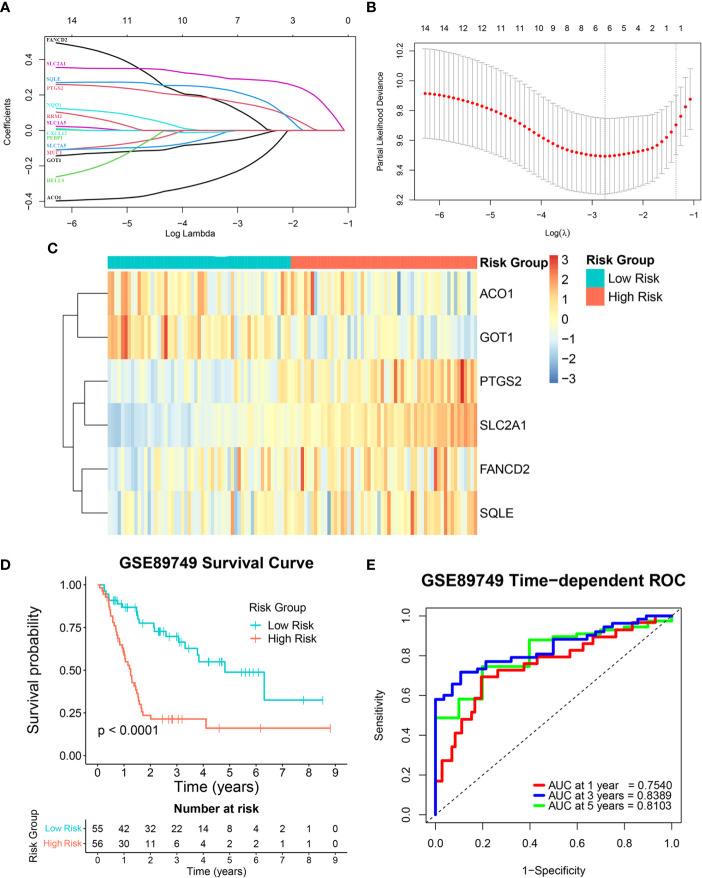
Construction of the ferroptosis-related gene (FRG) signature in a training cohort. **(A, B)** A least absolute shrinkage and selection operator (LASSO) Cox regression analysis of the candidate genes. **(C)** A heatmap showing the expression levels and distribution of the six-FRG signature. **(D)** A Kaplan-Meier curve showing the overall survival of CCA patients. **(E)** Area under the curve (AUC) of time-dependent receiver operating characteristic (ROC) curves showing the six-FRG signature’s predictive efficacy for survival time in CCA patients.

This study’s reliability was ensured by excluding three out of the 17 candidate genes (*MTIG*, *TF*, and *HAMP*) from gene signature construction. While these three genes were downregulated in tumor samples, their higher expression was associated with poorer prognosis. Therefore, the expression levels of the 14 remaining candidate genes and overall survival data from the GSE89749 cohort were used as a training cohort to construct the FRG signature *via* a LASSO Cox regression analysis. A six-FRG signature (*ACO1*, *GOT1*, *PTGS2*, *SLC2A1*, *FANCD2*, and *SQLE*) was identified based on the LASSO Cox regression with the minimum optimal lambda value using tenfold cross-validation (**Figures 3A, B**). Each patient’s risk score was calculated using the LASSO Cox regression analysis coefficient and patients were divided into low-risk and high-risk groups based on their median risk score. The six-FRG signature’s expression in the two risk groups was visualized as a heatmap (**Figure 3C**). Kaplan-Meier curves were used to compare survival in the two risk groups. They showed that survival was significantly longer in the low-risk group than in the high-risk group (*p*-value < 0.0001; **Figure 3D**). The six-FRG signature’s predictive efficacy was evaluated with a time-dependent ROC curve. The area under the ROC curves (AUCs) for the six-FRG signature at 1, 3 and 5 years of survival were 0.7540, 0.8389, and 0.8103, respectively, suggesting that it had high sensitivity and specificity (**Figure 3E**).

### 3.3 FRG signature validation in independent cohorts

**Figure 4:**
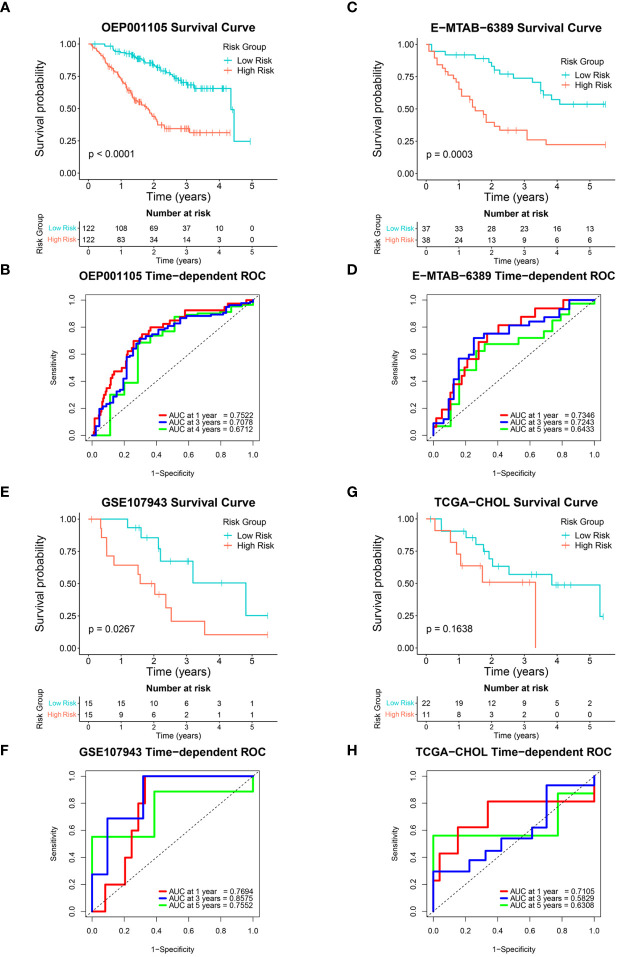
Validation of the six-FRG signature in four independent cohorts. **(A, C, E, G)** Kaplan-Meier curves and **(B, D, F, H)** time-dependent ROC curves for each independent cohort.

The six-FRG signature’s reproducibility was assessed using four independent cohorts. Patients with overall survival < 30 days were excluded from the validation cohorts. The model obtained with the training GSE89749 cohort was used to calculate each patient’s risk score in the validation cohorts. The OEP001105 (244 patients), E-MTAB-6389 (75 patients), GSE107943 (30 patients), and TCGA-CHOL (33 patients) cohorts were divided into low-risk and high-risk groups according to their median risk score, except for the TCGA-CHOL cohort, which was divided using best cut-off. Kaplan-Meier curves for the OEP001105, E-MTAB-6389, and GSE107943 cohorts indicated that survival was significantly shorter in the high-risk group than in the low-risk group (*p*-value < 0.0001, *p*-value = 0.0003, and *p*-value = 0.0267, respectively; **Figures 4A, C, E**). While the Kaplan-Meier curve for the TCGA-CHOL cohort was non-significant (*p*-value = 0.1638), this might reflect population variation due to small size. Nevertheless, the TCGA-CHOL cohort’s Kaplan-Meier curve indicated that the high-risk group tended to have shorter survival times than the low-risk group, which differed in their median survival (**Figure 4G**). ROC curves were used to assess the FRG signature’s accuracy in predicting patient survival. The AUCs at 1, 3, and 4 years were 0.7522, 0.7078, and 0.6712 in the OEP001105 cohort, respectively (**Figure 4B**). The AUCs at 1, 3, and 5 years were 0.7346, 0.7234, and 0.6433 in the E-MTAB-6389 cohort, respectively (**Figure 4D**). These results indicated that the FRG signature showed the greatest accuracy at one year, then gradually decreased with increasing years. However, the AUCs at 1, 3, and 5 years were 0.7694, 0.8575, and 0.7552 in the GSE107943 cohort, respectively, showing the greatest accuracy at three years (**Figure 4F**). Furthermore, the AUCs at 1, 3, and 5 years were 0.7105, 0.5829, and 0.6308, in the TCGA-CHOL cohort, respectively (**Figure 4H**). Altogether, these results suggested that our FRG signature could accurately predict CCA prognosis in patients.

### 3.4 Independent prognostic value of the six-FRG signature and clinical characteristics

**Figure 5:**
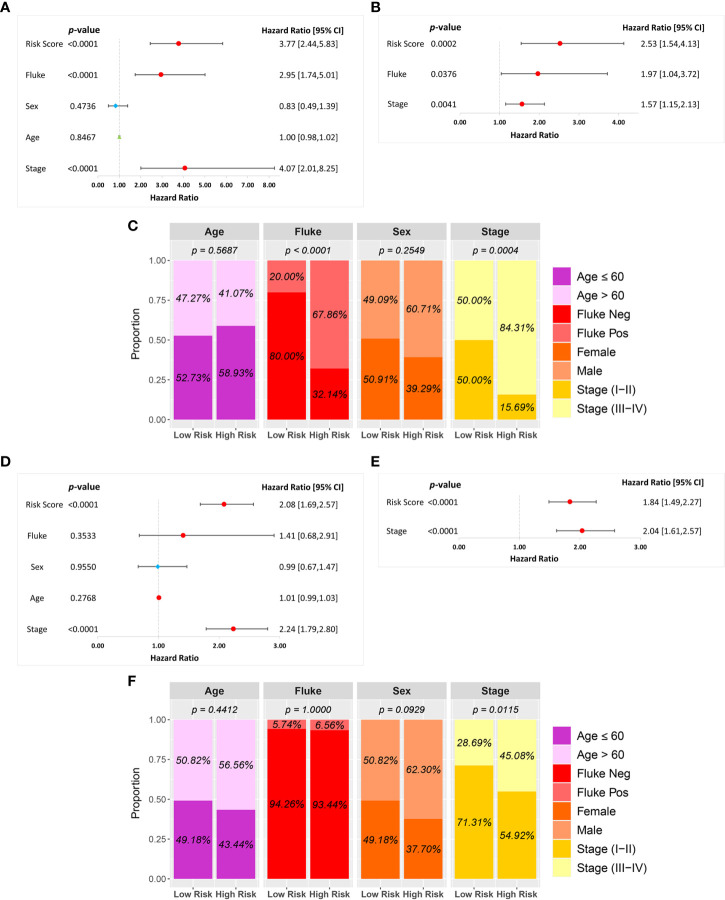
Univariate and multivariate Cox regression analyses of the six-FRG signature and other clinical characteristics in the **(A–C)** GSE89749 and **(D–F)** OEP00105 cohorts.

A univariate Cox regression analysis was used to investigate the association between risk score and clinical characteristics, including fluke infection, sex, age, and stage in the GSE89749 and OEP001105 cohorts. A forest plot of the univariate Cox regression showed that risk score (HR = 3.77), fluke infection (HR = 2.95), and stage (HR = 4.07) were significantly associated with patient survival in the GSE89749 cohort (**Figure 5A**). Similarly, risk score (HR = 2.08) and stage (HR = 2.24) were significantly associated with patient survival in the forest plot of OEP001105 cohort (**Figure 5D**). A multivariate Cox regression was performed to determine whether risk score was an independent prognostic factor. The forest plot for the multivariate Cox regression showed that risk scores were an independent prognostic factor in the GSE89749 and OEP001105 cohorts (*p*-value < 0.001; **Figures 5B, E**). Furthermore, the high-risk group was also associated with fluke infection and high clinical stages in the GSE89749 cohort, but only with high clinical stages in OEP001105 cohort (**Figures 5C, F**).

### 3.5 GO and KEGG pathway enrichment analyses

**Figure 6:**
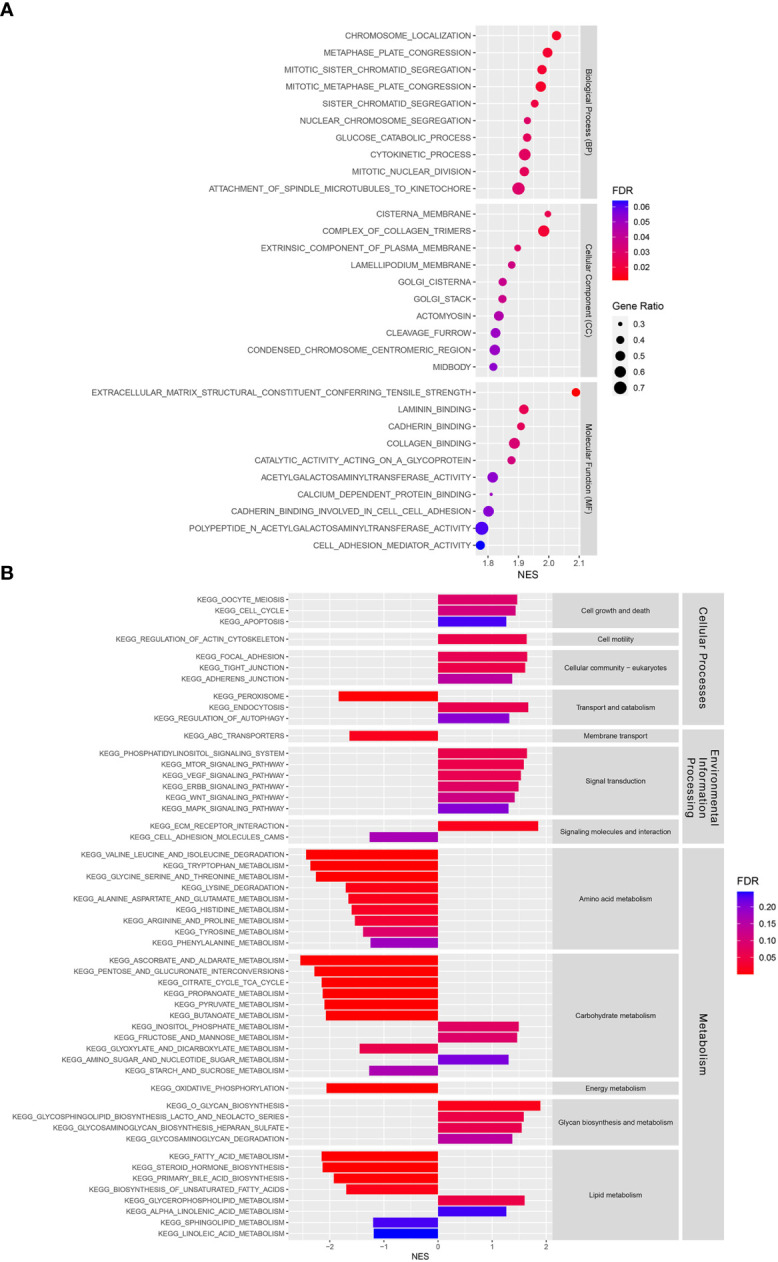
Functional and pathway enrichment analyses in a training cohort. **(A)** Gene Ontology (GO) analysis of differences between risk groups in three functional categories: Biological processes (BPs), cellular components (CCs), and molecular functions (MFs). **(B)** A Kyoto Encyclopedia of Genes and Genomes (KEGG) pathway enrichment analysis of differences between risk groups.

GSEA was performed to investigate the underlying differences in functions and biological processes between the low-risk and high-risk groups based on GO and KEGG pathways. The GO analysis identified biological process (BP), cellular component (CC), and molecular function (MF) enriched in the high-risk CCA patient groups (**Figure 6A**). KEGG pathway analysis identified differential pathway enrichment between the low-risk and high-risk CCA patient groups (**Figure 6B**).

### 3.6 Immune cell infiltration and TME analysis

**Figure 7:**
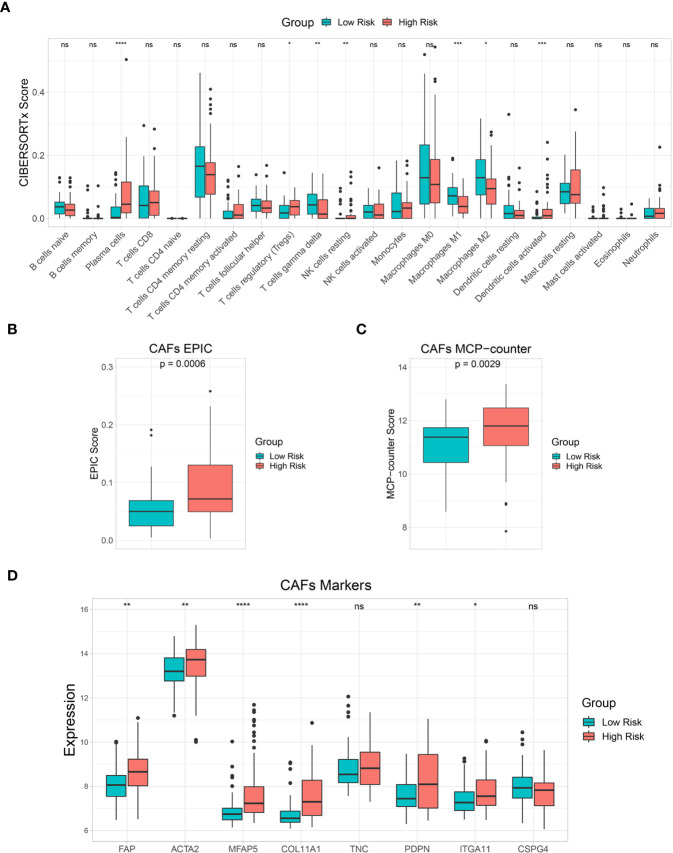
Immune cell infiltration and tumor microenvironment (TME) analysis. **(A)** A box plot showing the differences in multiple immune cells between low-risk and high-risk groups based on the CIBERSORTx algorithm. **(B, C)** A box plot showing differences in cancer-associated fibroblast (CAF) score based on EPIC and MCP-counter algorithms. **(D)** A box plot comparing CAF marker expression levels between risk groups. Key: ns, not significant; *p < 0.05; **p < 0.01; ***p < 0.001; ****p < 0.0001.

The 22 immune cell infiltration results estimated by the CIBERSORTx algorithm showed that plasma cells, regulatory T cells (Tregs), resting natural killer (NK) cells, and activated dendritic cells were significantly higher in the high-risk than in the low-risk groups. Gamma delta T cells and M1 and M2 macrophages were significantly lower in the high-risk group than in the low-risk group (**Figure 7A**). In addition, EPIC and MCP-counter algorithms were used to estimate CAF scores in the low-risk and high-risk groups. Both EPIC and MCP-counter algorithms showed that CAF scores were significantly higher in the high-risk group than in the low-risk group (**Figures 7B, C**). Furthermore, CAF specific marker expression was compared between the risk groups. *FAP*, *ACTA2*, *MFAP5*, *COL11A1*, *PDPN*, and *ITGA11* expression levels were significantly higher in the high-risk group than in the low-risk group (**Figure 7D**). These results indicated that immune responses and CAF statuses differed in these two patient groups, which might be translated to target the TME in CCA.

### 3.7 Potential drugs targeting the two risk groups and an immune checkpoint analysis

**Figure 8:**
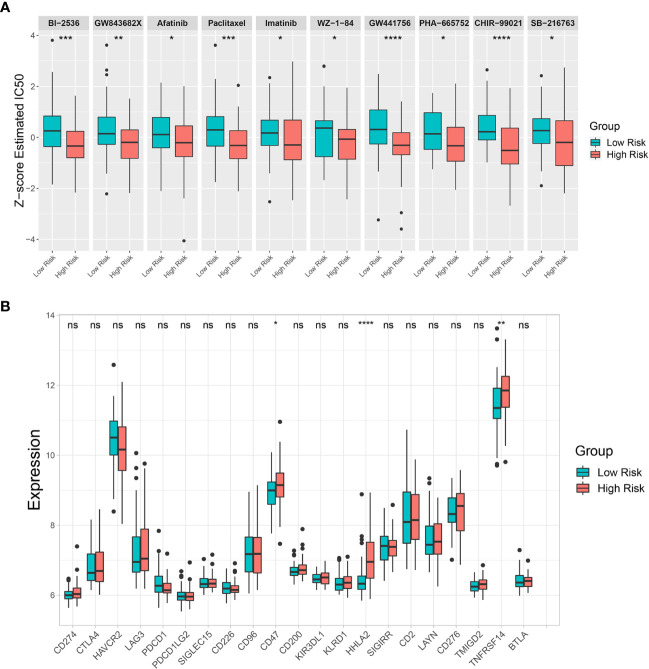
Drug sensitivity prediction and immune checkpoint analysis. **(A)** Differences in estimated half-maximal inhibitory concentration (IC_50_) between risk groups for 10 candidate drugs. **(B)** A box plot comparing immune checkpoint marker expression levels between risk groups. Key: ns, not significant; *p < 0.05; **p < 0.01; ***p < 0.001; ****p < 0.0001.

The GDSC database was used to estimate IC_50_ of various drugs *via* R’s “*pRRophetic*” package to predict drug sensitivity between the low-risk and high-risk groups. The estimated IC_50_ for 10 drugs (BI-2536, GW843682X, Afatinib, Paclitaxel, Imatinib, WZ-1-84, GW441756, PHA-665752, CHIR-99021, and SB-216763) out of 138 screened drugs were lower in the high-risk group than in the low-risk group, indicating that the high-risk group was more sensitive to these drugs than the low-risk group (**Figure 8A**). Moreover, an immune checkpoint analysis showed that *CD47*, *HHLA2*, and *TNFRSF14* expression was significantly higher in the high-risk group than in the low-risk group (**Figure 8B**). Therefore, these immune checkpoints might be effective immunotherapy targets in the high-risk CCA patient group.

### 3.8 Validation of the expression of six-FRG signature proteins in CCA patients

**Figure 9:**
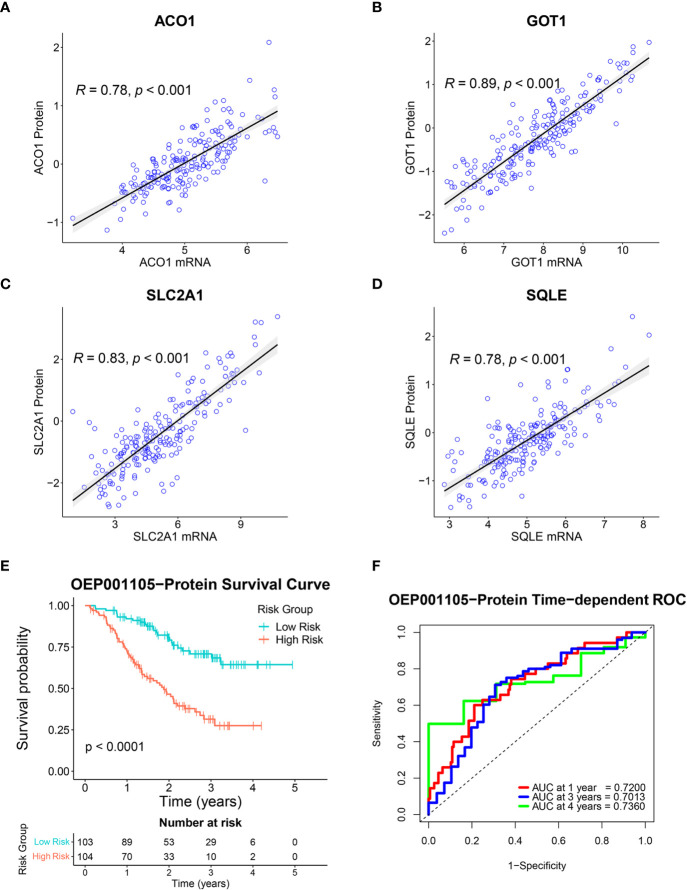
Validation of the six-FRG signature based on protein levels in the OEP001105 cohort. **(A–D)** Scatter plots showing the correlation between mRNA and protein expression levels of *ACO1*, *GOT1*, *SLC2A1*, and *SQLE*. **(E)** A Kaplan-Meier curve comparing survival time between patient groups defined on six-FRG signature protein levels. **(F)** Time-dependent ROC curves.

A total of 8,320 proteins were identified in the OEP001105 cohort. Four of six-FRG signature genes were present in this database (*ACO1*, *GOT1*, *SLC2A1*, and *SQLE*). Therefore, correlations between their mRNA and protein expression levels were analyzed in this cohort to confirm their protein expression. Protein and mRNA levels were significantly correlated for *ACO1* (*r* = 0.78), *GOT1* (*r* = 0.89), *SLC2A1* (*r* = 0.83) and *SQLE* (*r* = 0.78; **Figures 9A–D**). In addition, the score formula obtained from the training cohort was used to evaluate patient survival in this cohort based on the protein levels of these four genes. Kaplan-Meier curves showed that survival time was significantly shorter in the high-risk group than in the low-risk group (**Figure 9E**). The AUCs at 1, 3, and 4 years of survival were 0.7200, 0.7013, and 0.7360, respectively (**Figure 9F**). These results suggested that these four genes could provide an accurate prognostic signature. In addition, The Human Protein Atlas (HPA) database (https://www.proteinatlas.org/) (25) was used to confirm the protein expression of the six-FRG signature in CCA. The immunohistochemistry images from The HPA database showed that ACO1 and GOT1 levels were lower in tumor compared to normal tissues. In contrast, FANCD2, PTGS2, SLC2A1, and SQLE were higher in tumor compared to normal tissues (**Supplementary Figure S1**).

### 3.9 Expression of the six-FRG signature in CCA cell lines and ferroptosis inducer sensitivity

**Figure 10:**
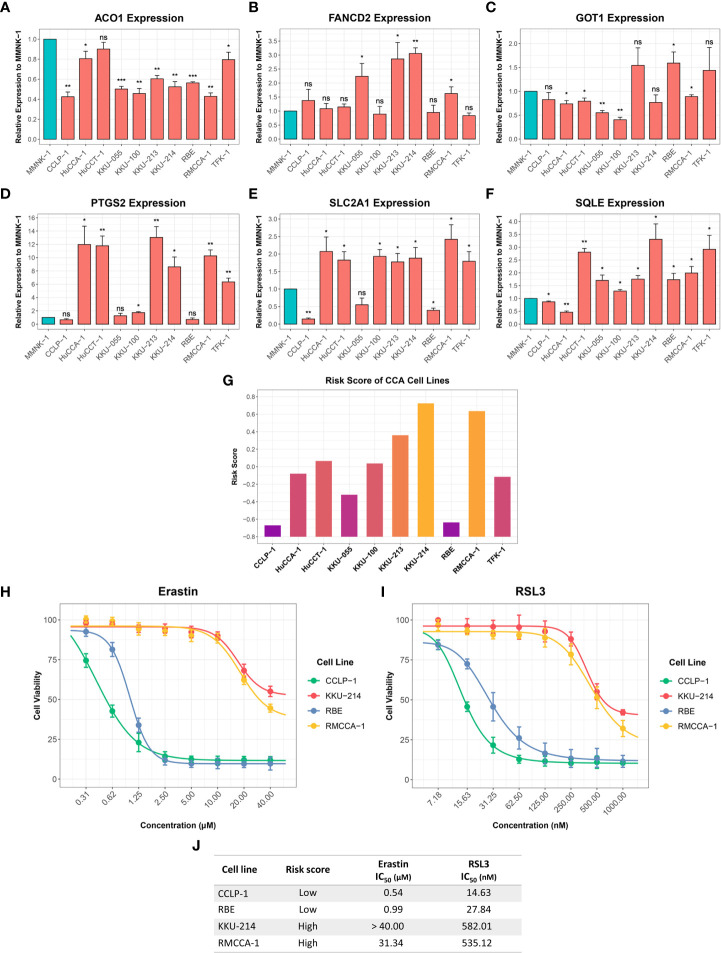
Expression of the six-FRG signature in CCA cell lines and their sensitivity to ferroptosis inducers. **(A-F)** The relative expression levels of the six-FRG signature in 10 CCA cell lines normalized to a non-tumor MMNK-1 cell line. **(G)** Risk scores calculated using six-FRG signature in 10 CCA cell lines. **(H)** Cell viability of CCLP-1, RBE, KKU-214, and RMCCA-1 treated with erastin. **(I)** Cell viability of CCLP-1, RBE, KKU-214, and RMCCA-1 treated with RSL3. **(J)** A table showing IC_50_ of erastin and RSL3 in CCLP-1, RBE, KKU-214, and RMCCA-1. Key: ns, not significant; *p < 0.05; **p < 0.01; ***p < 0.001.

RT-qPCR was used to investigate the expression of six-FRG signature in 10 CCA cell lines relative to a non-tumor cholangiocyte cell line (MMNK-1). *ACO1* and *GOT1* expression was downregulated in most CCA cell lines compared to the MMNK-1 cell line. In contrast, *FANCD2*, *PTGS2*, *SLC2A1*, and *SQLE* expression was upregulated in CCA cell line compared to the MMNK-1 cell line (**Figures 10A–F**). Interestingly, the expression of the six-FRG signature in CCA cell lines showed a similar trend to their expression in CCA tissues. Moreover, the formula constructed from the training cohort was used to calculate risk score for each CCA cell line and divided them into high-risk and low-risk groups. The functional role of the six-FRG signature in ferroptosis was investigated by examining the sensitivity of the two CCA cell lines with the highest (KKU-214 and RMCCA-1) and lowest (CCLP-1 and RBE) risk scores to ferroptosis inducers (**Figure 10G**). Their sensitivity to ferroptosis inducers erastin and RSL3 was compared using IC_50_ values. Interestingly, the two CCA cell lines with the lowest-risk scores (CCLP-1 and RBE) were more sensitive to both ferroptosis inducers. The CCA cell lines with the highest-risk scores (KKU-214 and RMCCA-1) had higher IC_50_ values than the CCA cell lines with the lowest-risk scores following treatment with both ferroptosis inducers (**Figures 10H–J**). These results highlight the association between the risk score and ferroptosis sensitivity. CCA cell lines with higher-risk scores were more resistant to ferroptosis, indicating that protective mechanisms against ferroptosis might be enhanced in such CCA cell lines.

## 4 Discussion

CCA is the second most common cancer in the hepatobiliary system. Patients with CCA have poor prognoses and high mortality rate in which patients in advanced stages have a low 5-year survival rate of 5% to 10%. Due to tumor heterogeneity and no effective therapy, patients with CCA have the worst prognosis. Targeting programmed cell death is one of the most effective cancer treatments, and dysregulation of this pathway and its-related genes are directly associated with prognosis of patients. Accumulating evidence has demonstrated that ferroptosis, a recently identified regulated cell death pathway, is a promising cancer therapy in several cancers. In addition, several studies have shown the relationship between FRGs and patient prognosis. While few previous studies have explored the relationship between FRG signatures and prognosis in CCA patients, a comprehensive analysis and validation in more patients and cohorts have not been performed. Moreover, to our knowledge, studies focusing on the role of prognostic genes and their associations with ferroptosis are limited. Consequently, how ferroptosis contributes to CCA remains unclear. Therefore, discovering a novel FRG signature might aid in predicting prognosis and developing novel therapeutic targets that can help to improve the overall survival of CCA patients.

This study constructed an FRG signature to predict the prognosis of CCA patients. Seventeen DEGs with prognostic values were identified in a training cohort using paired-sample *t*-tests and univariate Cox regression analyses. We then obtained a six-FRG signature related to patient survival in a LASSO Cox regression analysis in a training cohort. Following Kaplan-Meier analyses, our FRG signature showed that CCA patients with high-risk scores had poor prognoses, and low-risk scores had a longer survival time, indicating that our new FRG signature had strong prognostic potential in CCA patients. Predictive ability was further confirmed *via* ROC curve analyses, where our FRG signature had adequate predictive power. Furthermore, univariate and multivariate Cox regression analyses indicated that the risk score based on this six-FRG signature was an independent prognostic factor. The risk scores were associated with fluke infection and clinical stages in patients in the GSE89749 cohort, but only with clinical stages in patients in the OEP001105 cohort, potentially reflecting the small number of fluke- infected patients in this cohort. Therefore, our novel six-FRG signature can accurately predict prognoses for CCA patients and divide them into low-risk and high-risk groups in which appropriate therapeutic strategies can be used for personalized therapy.

High expression of four genes in the six-FRG signature (*FANCD2*, *PTGS2*, *SLC2A1*, and *SQLE*) were associated with poor prognosis in CCA patients and low expression of the six-FRG other two genes (*ACO1* and *GOT1*) were associated with poor prognosis in CCA patients. Previous studies have shown that most FRGs in the signature are involved in tumor progression in CCA and various other cancers. *ACO1*, also known as *IRP1*, is an RNA-binding protein that controls iron homeostasis by regulating *TFRC* and *FTH1* expression in CCA and hepatocellular carcinoma (26, 27). *ACO1* depletion decreased iron levels and suppressed erastin- and RSL3-induced ferroptosis in melanoma (28). Moreover, *GOT1* downregulation suppressed ferroptosis in melanoma by reducing α-ketoglutarate which is a metabolic intermediate in ROS production (29). However, the role of *GOT1* in CCA remains unknown. In contrast, *FANCD2* was found to be a ferroptosis suppressor in bone marrow injury by regulating the expression of iron metabolism and lipid peroxidation-related genes (30). *FANCD2* was identified as a prognostic gene associated with poor prognosis in colon cancer, lung adenocarcinoma, clear cell renal cell carcinoma, and glioma (31–34). *PTGS2*, also known as *COX-2*, was identified as an upregulated ferroptosis marker during ferroptosis (11). *PTGS2* was upregulated in tumor tissues and promoted tumor progression and chemotherapy resistance in various cancers (35–37). *PTGS2* has been shown to promote tumor development in CCA, while its inhibition potentiated conventional chemotherapy effects (38–41). *SLC2A1*, also known as *GLUT1*, is a glucose transporters family member. *SLC2A1* was found to be upregulated and play a role in tumor progression in various cancers (42–45). A previous study identified *SLC2A1* as a prognostic gene in CCA patients (46, 47). Overexpression of *SQLE*, a key enzyme in cholesterol biosynthesis, increased lipid peroxidation, leading to ferroptosis (48). Recent studies found that *SQLE* promoted tumor progression, and its expression was associated with poor prognosis in breast cancer, pancreatic adenocarcinoma, and bladder cancer (49–51). In summary, consistent with the previous studies, our study showed that *ACO1* and *GOT1* were downregulated and correlated with good prognosis in CCA patients. In contrast, *FANCD2*, *PTGS2*, *SLC2A1*, and *SQLE* were upregulated and correlated with poor prognosis in CCA patients. However, their functional roles in ferroptosis and CCA progression remain unknown, and mechanistic studies on each prognostic FRG in our signature are needed.

In addition, our GO analysis identified BP, CC, and MF enrichments in the high-risk group. Among the BPs, the nuclear division process was enriched in the high-risk group, indicating that they might have higher proliferation than the low-risk group. The CC results showed that Golgi apparatus components were enriched in the high-risk group. Previous studies have shown that the Golgi apparatus plays important roles in cellular redox control and prevents ferroptosis (52, 53). The MF results showed that cell adhesion molecules, such as laminin and cadherin binding, and cell adhesion mediator activity were enriched in the high-risk group. Recently, cell-cell interaction was found to regulate ferroptosis sensitivity (54). Moreover, a KEGG pathway analysis was used to identify the main signaling pathways contributing to CCA by comparing low-risk and high-risk groups. Many signal transduction pathways frequently dysregulated in cancers were enriched in the high-risk group, including the phosphatidylinositol, mammalian target of rapamycin (mTOR), vascular endothelial growth factor (VEGF), erb-be receptor tyrosine kinase (ERBB), Wnt, and mitogen-activated protein kinase (MAPK) signaling pathways. These signaling pathways have been shown to play important roles in CCA and various cancers (55–66). In contrast, amino acid, carbohydrate, and lipid metabolism were enriched in the low-risk group, while glycan biosynthesis and metabolism were found to be enriched in the high-risk group. In summary, our study has shown differences in signaling pathway enrichment between CCA risk groups, which could be targeted to develop treatment strategies and improve prognosis in each CCA risk group.

Accumulating evidence has shown the interplay between tumor cells and other cell types in the TME, including a subset of immune cells and CAFs that play important roles in tumor progression and developing therapeutic resistance (67). CCA is a dysmorphic tumor with abundance CAFs and immunosuppressive cells in the TME (68). Therefore, we further investigated immune cell and CAF enrichment by comparing low-risk and high-risk groups. Tregs are an immune system component that suppressed anticancer immunity and are associated with poor prognoses in various cancers (68–71). Recent studies showed that FOXP3+, a Treg marker associated with poor prognosis in CCA patients (72, 73). Plasma cells were found to be a source of immunosuppressive factor interleukin (IL)-10 (74, 75). Moreover, CAFs are major TME components with tumorigenic properties, especially, in immunosuppressive TME modulation (76). This study found that levels of these immunosuppressive components, including Tregs, plasma cells, and CAFs were significantly higher in the high-risk group. Therefore, immunosuppressive components might be promising therapeutic targets to improve the efficacy of CCA treatment and patient survival.

Drug sensitivity prediction analysis was performed to overcome treatment resistance and improve CCA patient prognosis, particularly in the high-risk group. Our analysis predicted 10 effectives potentially candidate drugs for the high-risk CCA patient group. Paclitaxel, Imatinib, and Afatinib are US Food and Drug Administration (FDA)-approved drugs used in clinics to treat various cancers. A recent study showed that Paclitaxel could be a drug candidate for CCA patients resistant to conventional therapies (77). Our analysis supported Paclitaxel potentially having a better effect in treating CCA patients within the high-risk group. The ABL, KIT, and PDGFR inhibitor Imatinib was effective in treating gastrointestinal stromal tumor with *KIT* or *PDGFR* overexpression or mutation (78). The epidermal growth factor receptor (EGFR) and ERBB tyrosine kinase inhibitor Afatinib has been used as a first-line drug for non-small cell lung cancer patients with an *EGFR* mutation (79). However, relatively few studies have investigated the efficacy of these kinase inhibitors in CCA patients (80–82). Moreover, PLK1 inhibitors BI-2536 and GW843682X were predicted to be effective drugs for the high-risk group. Consistent with our results, previous studies have shown that PLK1 was associated with poor prognosis in CCA patients, and its inhibition was effective against CCA cells (83–85). *NTRK1* fusion has been found in CCA patients (86) resulting in constitutive *TRKA* activation, and its inhibition showed positive responses in specific CCA patient groups (87). In this study, a selective TRKA inhibitor GW441756 was shown more effective in the high-risk group. In addition, selective c-Met inhibitor PHA-665752 and GSK3B inhibitors CHIR-99021 and SB-216763 were shown to be more effective in the high-risk group. Both c-MET and GSK3B were associated with poor prognosis, and targeting c-MET or GSK3B has been reported to be potentially effective in treating CCA patients (88–93). Altogether, based on risk stratification groups, our study predicted effective candidate drugs for treating CCA patients, which could be used in precision therapy to improve their prognosis.

Immunotherapy has been proposed as a potential therapeutic option for CCA (94, 95). However, the outcomes of anti-PD-1/PD-L1 immune checkpoint inhibitors (ICIs) remain controversial in CCA. In this study, *PD-1* (*PDCD-1*) and *PD-L1* (*CD274*) expression levels did not differ between low-risk and high-risk groups. Interestingly, other immune checkpoint markers, including *CD47*, *HHLA2*, and *TNFRSF14* showed significantly higher expression in the high-risk group. Therefore, these immune checkpoints might be potential targets for developing ICIs to reactivate antitumor immunity which might improve the survival of patients with poor prognoses.

Previous studies have analyzed FRG signatures in several cancers. However, most have analyzed FRG signatures and classified patients into low-risk and high-risk groups that did not reflect relative ferroptosis levels (96–98). Therefore, this study calculated risk scores based on the expression levels of a six-FRG signature in each CCA cell line and could divide them into low-risk and high-risk groups. Using two representative CCA cell lines of the low-risk and high-risk groups, our study showed that low-risk score CCA cell lines were significantly more sensitive to ferroptosis inducers than high-risk score CCA cell lines. This result suggests that ferroptosis resistance might explain the relationship between the high-risk group and poor prognosis. Consistent with our findings, a previous study showed that activating ferroptosis suppressor genes enabled CCA cells to evade ferroptosis (99). Therefore, inhibiting ferroptosis suppressor genes and targeting ferroptosis resistance mechanisms might be effective therapeutic strategies for improving prognosis, particularly in the high-risk group. Besides ferroptosis resistance mechanisms, other factors, such as CCA stage, might contribute to differences in ferroptosis inducer sensitivity in the two CCA cell line groups. Unfortunately, information on the stages of CCA cell lines is only available for the high-risk group (i.e., RMCCA-1: T2N0M0; KKU-214: stage IVB)

However, our study had some limitation. First, the data used in this study were collected from public databases. Consequently, they differed appreciably in their CCA patient numbers, patient heterogeneity, ethnicity, and etiology. Second, the FRGs were identified from FerrDb and previous studies. However, the role of ferroptosis in cancers is still in its infancy. Therefore, some unidentified FRGs might be missing from our analyses. Finally, further *in vitro* and *in vivo* studies are needed to investigate the roles of these prognostic genes in ferroptosis and their underlying mechanisms in CCA.

## 5 Conclusions

In summary, our study showed that a six-FRG signature scoring model could divide CCA patients into low-risk and high-risk groups. Our novel FRG signature model effective predicted the prognosis of CCA patients, potentially providing prognostic biomarkers and potential therapeutic targets for CCA patients. Moreover, oncogenic signaling pathways, immune cell and CAF infiltration, drug sensitivity, and immune checkpoint marker expression differed between two CCA risk groups, which might represent novel therapeutic targets for improving survival, particularly of patients with poor prognoses. In addition, we predicted drug candidates for CCA patients in the high-risk group. Based on ferroptosis sensitivity in low-risk and high-risk CCA cell lines, our results suggest that ferroptosis resistance is associated with the high-risk group and targeting ferroptosis resistance mechanisms in this group could be a promising therapeutic strategy for these CCA patients.

## Data availability statement

The datasets presented in this study can be found in online repositories. The names of the repository/repositories and accession number(s) can be found in the article/**Supplementary Material**.

## Author contributions

AS-F collected data, performed all *in vitro* experiments, analyzed statistics and bioinformatics, organized the figures and drafted the manuscript under the supervision of SJ. AM served as the mentor of SJ for the Research Grant for New Scholars and participated in the editing of this manuscript. SJ conceived and designed the study; analyzed and interpreted the data; participated in the writing and editing of this manuscript. All authors contributed to the article and approved the submitted version.
