# Supplementary material for: Identification and validation of a novel ferroptosis-related gene signature for prognosis and potential therapeutic target prediction in cholangiocarcinoma
Source: Front Immunol. 2023 Jan 17;13:1051273. doi: 10.3389/fimmu.2022.1051273 (PMC9887182; doi:10.3389/fimmu.2022.1051273)
Supplement: Supplementary file 1 [file Presentation_1.pptx]

## Slide 1
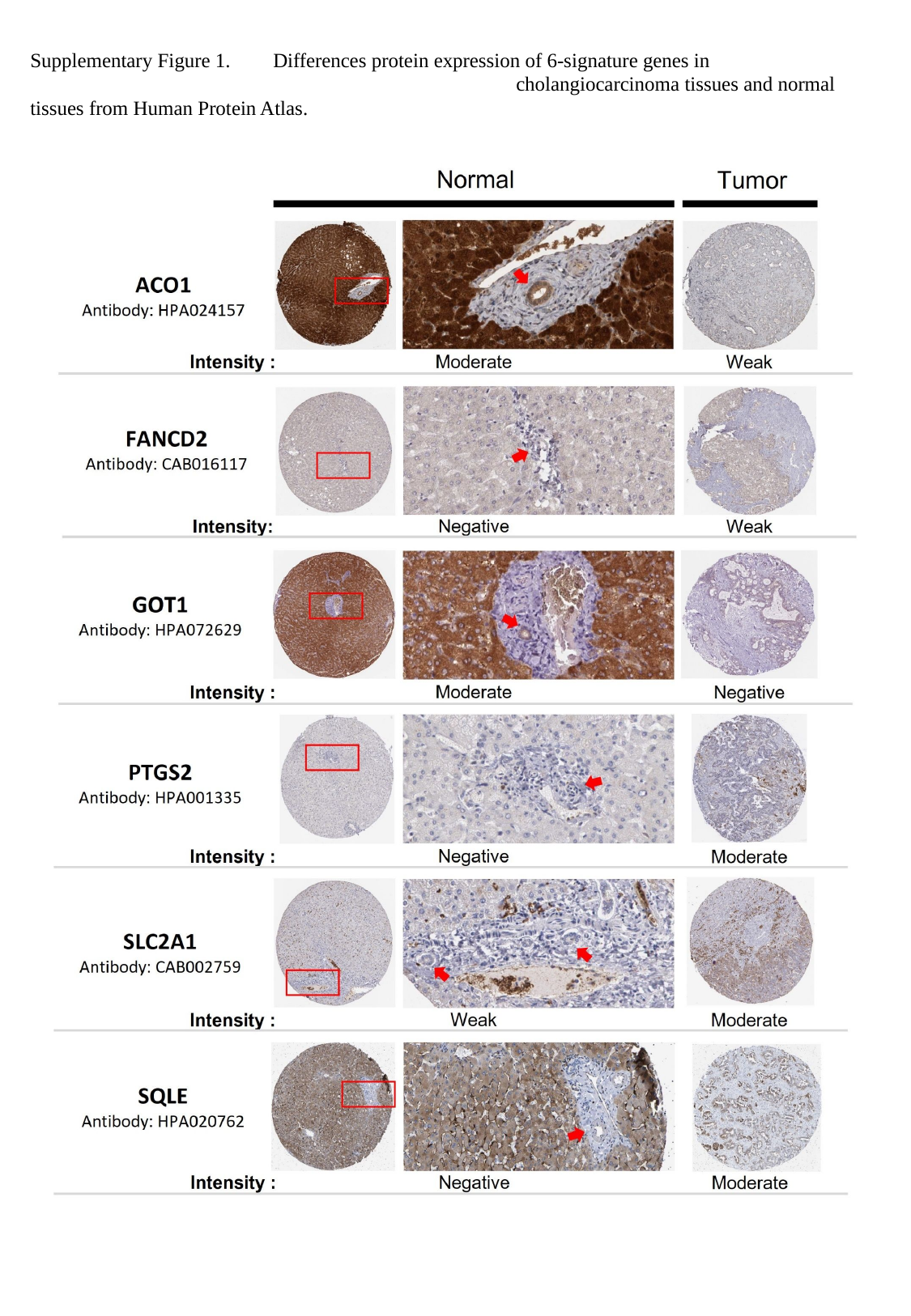

Supplementary Figure 1.	Differences protein expression of 6-signature genes in 						cholangiocarcinoma tissues and normal tissues from Human Protein Atlas.

## Slide 2
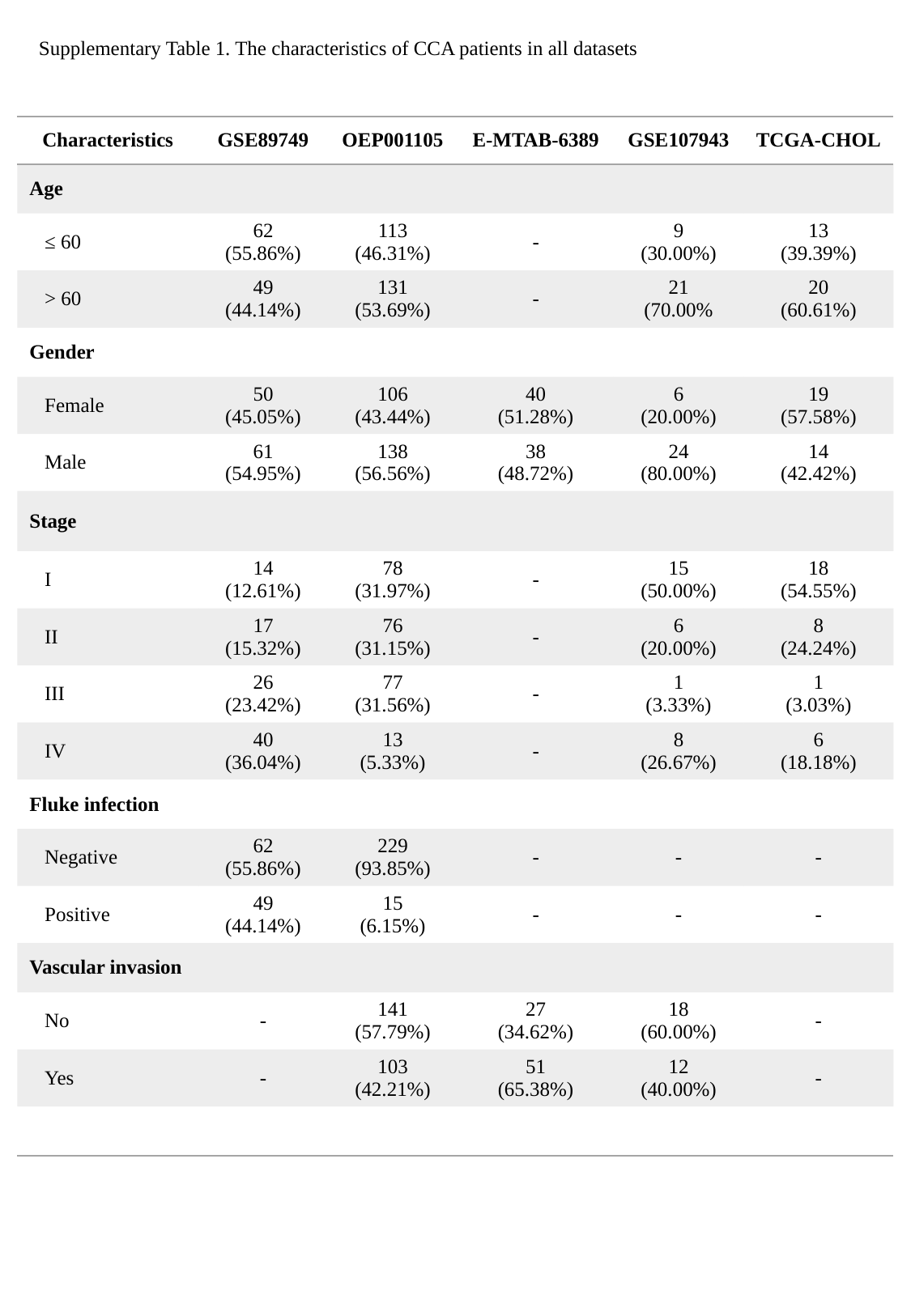

Supplementary Table 1. The characteristics of CCA patients in all datasets
| Characteristics | GSE89749 | OEP001105 | E-MTAB-6389 | GSE107943 | TCGA-CHOL |
| --- | --- | --- | --- | --- | --- |
| Age | | | | | |
| ≤ 60 | 62 (55.86%) | 113 (46.31%) | - | 9 (30.00%) | 13 (39.39%) |
| > 60 | 49 (44.14%) | 131 (53.69%) | - | 21 (70.00% | 20 (60.61%) |
| Gender | | | | | |
| Female | 50 (45.05%) | 106 (43.44%) | 40 (51.28%) | 6 (20.00%) | 19 (57.58%) |
| Male | 61 (54.95%) | 138 (56.56%) | 38 (48.72%) | 24 (80.00%) | 14 (42.42%) |
| Stage | | | | | |
| I | 14 (12.61%) | 78 (31.97%) | - | 15 (50.00%) | 18 (54.55%) |
| II | 17 (15.32%) | 76 (31.15%) | - | 6 (20.00%) | 8 (24.24%) |
| III | 26 (23.42%) | 77 (31.56%) | - | 1 (3.33%) | 1 (3.03%) |
| IV | 40 (36.04%) | 13 (5.33%) | - | 8 (26.67%) | 6 (18.18%) |
| Fluke infection | | | | | |
| Negative | 62 (55.86%) | 229 (93.85%) | - | - | - |
| Positive | 49 (44.14%) | 15 (6.15%) | - | - | - |
| Vascular invasion | | | | | |
| No | - | 141 (57.79%) | 27 (34.62%) | 18 (60.00%) | - |
| Yes | - | 103 (42.21%) | 51 (65.38%) | 12 (40.00%) | - |
| | | | | | |

## Slide 3
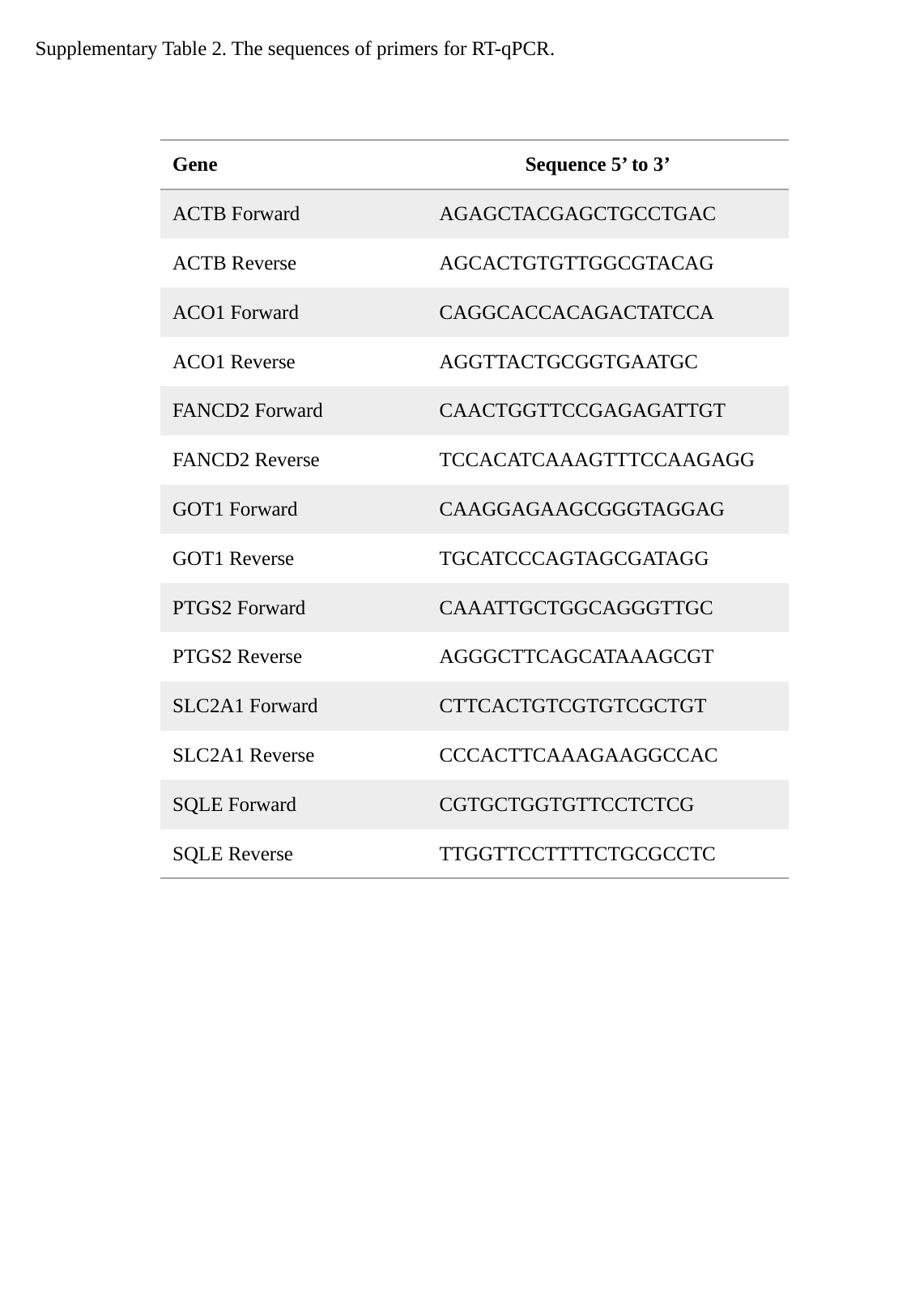

Supplementary Table 2. The sequences of primers for RT-qPCR.
| Gene | Sequence 5’ to 3’ |
| --- | --- |
| ACTB Forward | AGAGCTACGAGCTGCCTGAC |
| ACTB Reverse | AGCACTGTGTTGGCGTACAG |
| ACO1 Forward | CAGGCACCACAGACTATCCA |
| ACO1 Reverse | AGGTTACTGCGGTGAATGC |
| FANCD2 Forward | CAACTGGTTCCGAGAGATTGT |
| FANCD2 Reverse | TCCACATCAAAGTTTCCAAGAGG |
| GOT1 Forward | CAAGGAGAAGCGGGTAGGAG |
| GOT1 Reverse | TGCATCCCAGTAGCGATAGG |
| PTGS2 Forward | CAAATTGCTGGCAGGGTTGC |
| PTGS2 Reverse | AGGGCTTCAGCATAAAGCGT |
| SLC2A1 Forward | CTTCACTGTCGTGTCGCTGT |
| SLC2A1 Reverse | CCCACTTCAAAGAAGGCCAC |
| SQLE Forward | CGTGCTGGTGTTCCTCTCG |
| SQLE Reverse | TTGGTTCCTTTTCTGCGCCTC |

## Slide 4
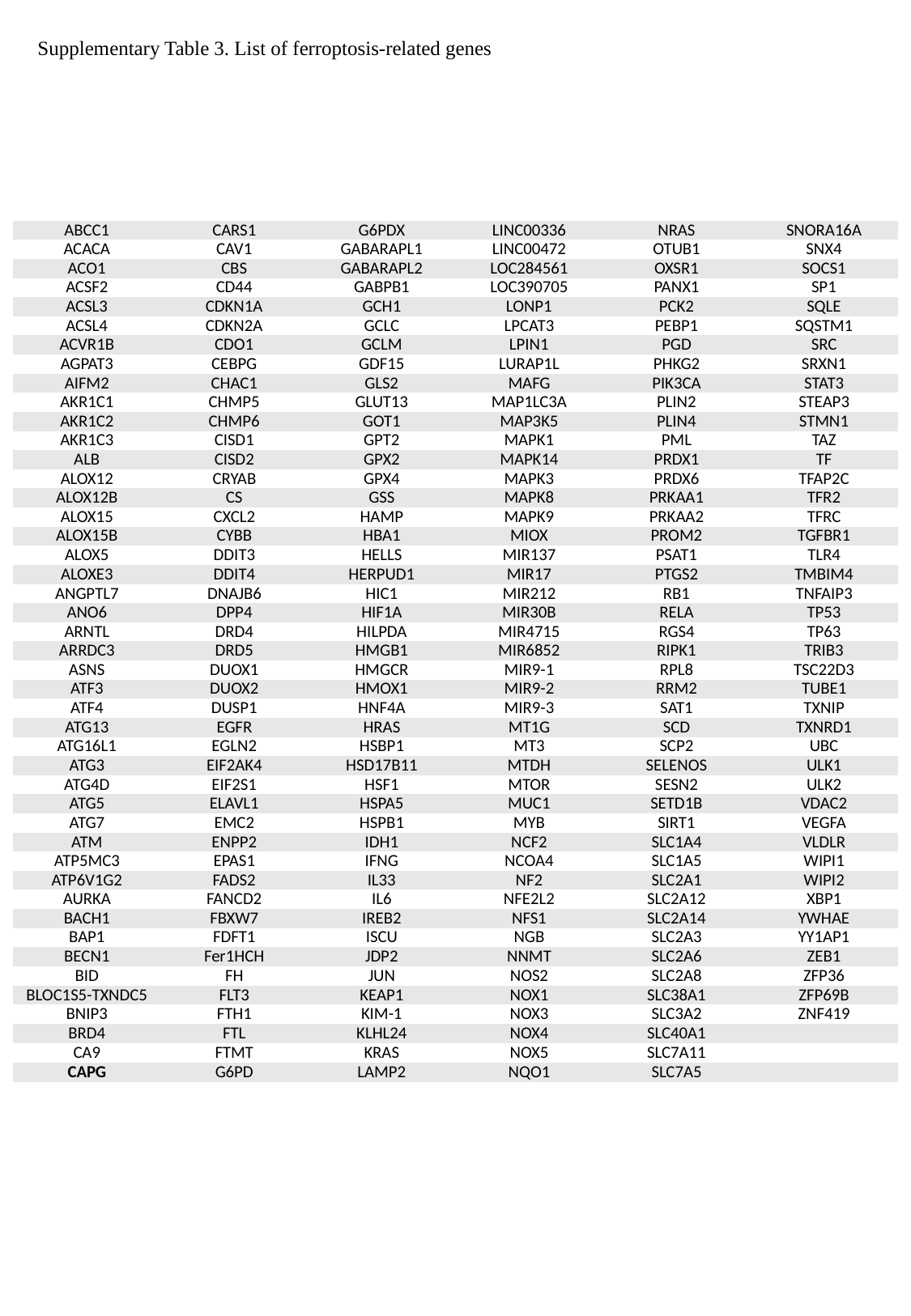

Supplementary Table 3. List of ferroptosis-related genes
| ABCC1 | CARS1 | G6PDX | LINC00336 | NRAS | SNORA16A |
| --- | --- | --- | --- | --- | --- |
| ACACA | CAV1 | GABARAPL1 | LINC00472 | OTUB1 | SNX4 |
| ACO1 | CBS | GABARAPL2 | LOC284561 | OXSR1 | SOCS1 |
| ACSF2 | CD44 | GABPB1 | LOC390705 | PANX1 | SP1 |
| ACSL3 | CDKN1A | GCH1 | LONP1 | PCK2 | SQLE |
| ACSL4 | CDKN2A | GCLC | LPCAT3 | PEBP1 | SQSTM1 |
| ACVR1B | CDO1 | GCLM | LPIN1 | PGD | SRC |
| AGPAT3 | CEBPG | GDF15 | LURAP1L | PHKG2 | SRXN1 |
| AIFM2 | CHAC1 | GLS2 | MAFG | PIK3CA | STAT3 |
| AKR1C1 | CHMP5 | GLUT13 | MAP1LC3A | PLIN2 | STEAP3 |
| AKR1C2 | CHMP6 | GOT1 | MAP3K5 | PLIN4 | STMN1 |
| AKR1C3 | CISD1 | GPT2 | MAPK1 | PML | TAZ |
| ALB | CISD2 | GPX2 | MAPK14 | PRDX1 | TF |
| ALOX12 | CRYAB | GPX4 | MAPK3 | PRDX6 | TFAP2C |
| ALOX12B | CS | GSS | MAPK8 | PRKAA1 | TFR2 |
| ALOX15 | CXCL2 | HAMP | MAPK9 | PRKAA2 | TFRC |
| ALOX15B | CYBB | HBA1 | MIOX | PROM2 | TGFBR1 |
| ALOX5 | DDIT3 | HELLS | MIR137 | PSAT1 | TLR4 |
| ALOXE3 | DDIT4 | HERPUD1 | MIR17 | PTGS2 | TMBIM4 |
| ANGPTL7 | DNAJB6 | HIC1 | MIR212 | RB1 | TNFAIP3 |
| ANO6 | DPP4 | HIF1A | MIR30B | RELA | TP53 |
| ARNTL | DRD4 | HILPDA | MIR4715 | RGS4 | TP63 |
| ARRDC3 | DRD5 | HMGB1 | MIR6852 | RIPK1 | TRIB3 |
| ASNS | DUOX1 | HMGCR | MIR9-1 | RPL8 | TSC22D3 |
| ATF3 | DUOX2 | HMOX1 | MIR9-2 | RRM2 | TUBE1 |
| ATF4 | DUSP1 | HNF4A | MIR9-3 | SAT1 | TXNIP |
| ATG13 | EGFR | HRAS | MT1G | SCD | TXNRD1 |
| ATG16L1 | EGLN2 | HSBP1 | MT3 | SCP2 | UBC |
| ATG3 | EIF2AK4 | HSD17B11 | MTDH | SELENOS | ULK1 |
| ATG4D | EIF2S1 | HSF1 | MTOR | SESN2 | ULK2 |
| ATG5 | ELAVL1 | HSPA5 | MUC1 | SETD1B | VDAC2 |
| ATG7 | EMC2 | HSPB1 | MYB | SIRT1 | VEGFA |
| ATM | ENPP2 | IDH1 | NCF2 | SLC1A4 | VLDLR |
| ATP5MC3 | EPAS1 | IFNG | NCOA4 | SLC1A5 | WIPI1 |
| ATP6V1G2 | FADS2 | IL33 | NF2 | SLC2A1 | WIPI2 |
| AURKA | FANCD2 | IL6 | NFE2L2 | SLC2A12 | XBP1 |
| BACH1 | FBXW7 | IREB2 | NFS1 | SLC2A14 | YWHAE |
| BAP1 | FDFT1 | ISCU | NGB | SLC2A3 | YY1AP1 |
| BECN1 | Fer1HCH | JDP2 | NNMT | SLC2A6 | ZEB1 |
| BID | FH | JUN | NOS2 | SLC2A8 | ZFP36 |
| BLOC1S5-TXNDC5 | FLT3 | KEAP1 | NOX1 | SLC38A1 | ZFP69B |
| BNIP3 | FTH1 | KIM-1 | NOX3 | SLC3A2 | ZNF419 |
| BRD4 | FTL | KLHL24 | NOX4 | SLC40A1 | |
| CA9 | FTMT | KRAS | NOX5 | SLC7A11 | |
| CAPG | G6PD | LAMP2 | NQO1 | SLC7A5 | |
